# Potential public health impact of RTS,S malaria candidate vaccine in sub-Saharan Africa: a modelling study

**DOI:** 10.1186/s12936-015-1046-z

**Published:** 2015-12-23

**Authors:** Christophe J. Sauboin, Laure-Anne Van Bellinghen, Nicolas Van De Velde, Ilse Van Vlaenderen

**Affiliations:** GSK Vaccines, Avenue Fleming 20, 1300 Wavre, Belgium; CHESS in Health, Zwarte Leeuwstraat 69, 2820 Bonheiden, Belgium

**Keywords:** Malaria, Vaccination, RTS,S candidate vaccine, Public health

## Abstract

**Background:**

Adding malaria vaccination to existing interventions could help to reduce the health burden due to malaria. This study modelled the potential public health impact of the RTS,S candidate malaria vaccine in 42 malaria-endemic countries in sub-Saharan Africa.

**Methods:**

An individual-based Markov cohort model was constructed with three categories of malaria transmission intensity and six successive malaria immunity levels. The cycle time was 5 days. Vaccination was assumed to reduce the risk of infection, with no other effects. Vaccine efficacy was assumed to wane exponentially over time. Malaria incidence and vaccine efficacy data were taken from a Phase III trial of the RTS,S vaccine with 18 months of follow-up (NCT00866619). The model was calibrated to reproduce the malaria incidence in the control arm of the trial in each transmission category and published age distribution data. Individual-level heterogeneity in malaria exposure and vaccine protection was accounted for. Parameter uncertainty and variability were captured by using stochastic model transitions. The model followed a cohort from birth to 10 years of age without malaria vaccination, or with RTS,S malaria vaccination administered at age 6, 10 and 14 weeks or at age 6, 7-and-a-half and 9 months. Median and 95 % confidence intervals were calculated for the number of clinical malaria cases, severe cases, malaria hospitalizations and malaria deaths expected to be averted by each vaccination strategy. Univariate sensitivity analysis was conducted by varying the values of key input parameters.

**Results:**

Vaccination assuming the coverage of diphtheria-tetanus-pertussis (DTP3) at age 6, 10 and 14 weeks is estimated to avert over five million clinical malaria cases, 119,000 severe malaria cases, 98,600 malaria hospitalizations and 31,000 malaria deaths in the 42 countries over the 10-year period. Vaccination at age 6, 7-and-a-half and 9 months with 75 % of DTP3 coverage is estimated to avert almost 12.5 million clinical malaria cases, 250,000 severe malaria cases, 208,000 malaria hospitalizations and 65,400 malaria deaths in the 42 countries. Univariate sensitivity analysis indicated that for both vaccination strategies, the parameters with the largest impact on the malaria mortality estimates were waning of vaccine efficacy and malaria case-fatality rate.

**Conclusions:**

Addition of RTS,S malaria vaccination to existing malaria interventions is estimated to reduce substantially the incidence of clinical malaria, severe malaria, malaria hospitalizations and malaria deaths across 42 countries in sub-Saharan Africa.

**Electronic supplementary material:**

The online version of this article (doi:10.1186/s12936-015-1046-z) contains supplementary material, which is available to authorized users.

## Background

The health burden attributable to malaria is immense. The World Health Organization (WHO) estimated that in 2013 there were 163 million malaria cases and 528,000 malaria deaths in the WHO Africa Region [1]. In the updated Global Burden of Disease study, malaria was the second leading cause of disability-adjusted life-years (DALYs) in sub-Saharan Africa and the first cause in western Africa in 2013 [2]. Most malaria deaths occur in children aged <5 years (estimated 437,000 malaria deaths in WHO Africa Region in 2013) [1]. Among children aged <5 years (excluding infants in the first month of life), malaria was the leading cause of death in Africa in 2010, accounting for 15 % of deaths [3].

Current preventive interventions in malaria aim to reduce malaria transmission by insect vectors (insecticide spraying and the use of insecticide-treated bed nets), or to reduce the disease burden by prophylactic treatment of defined population groups such as pregnant women, infants or children [4, 5]. The main treatment for malaria is artemisinin-based combination therapy [6]. Decreases in estimated malaria case incidence (34 % decrease between 2000 and 2013) and estimated malaria mortality rate in children aged <5 years (58 % decrease between 2000 and 2013) in the WHO Africa region have been reported, and probably reflect the combined effects of malaria intervention programmes, increased urbanization and overall economic development [1]. However, current malaria interventions have some limitations. Insecticide resistance has been reported in 49/63 reporting countries worldwide since 2010, and artemisinin-resistant *Plasmodium falciparum* malaria has been reported in five countries in Southeast Asia [1]. Insecticide-treated nets need to be routinely distributed and replaced when they wear out in order to maintain coverage and therefore effectiveness. A slower growth in insecticide-treated bed net coverage in 2012–2013 in sub-Saharan Africa was associated with a slower decline in malaria mortality between 2011 and 2013 [1].

A malaria vaccine could be a valuable addition to existing malaria control interventions. The RTS,S malaria candidate vaccine is a pre-erythrocytic vaccine, directed against a protein from the sporozoite stage of the *P. falciparum* malaria parasite. It aims to trigger an immune defence to prevent the parasites infecting the liver and red blood cells. The RTS,S malaria candidate vaccine is administered in three doses given at one-month intervals, with a possible booster dose given 18 months after the third dose. A double-blind randomized controlled Phase III trial in seven countries in Africa enrolled 15,460 infants aged 6–12 weeks and children aged 5–17 months between March 2009 and January 2011, and randomized them in a 1:1:1 ratio to receive the RTS,S candidate vaccine with a booster, without a booster, or a non-malaria comparator vaccine [7–9]. Results with 18 months of follow-up from the third dose of vaccine were published in 2014, combining the first two groups and comparing them with the third (control) group [10]. In children, vaccination reduced clinical malaria cases by 46 % [95 % confidence interval (CI) 42, 50 %], severe malaria cases by 34 % (95 % CI 15, 48 %) and malaria hospitalizations by 41 % (95 % CI 30, 50 %). In infants, the reduction in clinical malaria cases was 27 % (95 % CI 20, 32 %), with no significant protection against severe malaria or malaria hospitalization [10]. The highest number of malaria episodes averted was seen in areas with greatest malaria incidence [10].

The objective of the present analysis is to apply these 18-month clinical trial results to estimate the potential public health impact of the RTS,S candidate malaria vaccine on the number of clinical malaria cases, severe malaria cases, hospitalizations and deaths in malaria-endemic countries in sub-Saharan Africa using a cohort model.

## Methods

### Model structure

**Fig. 1:**
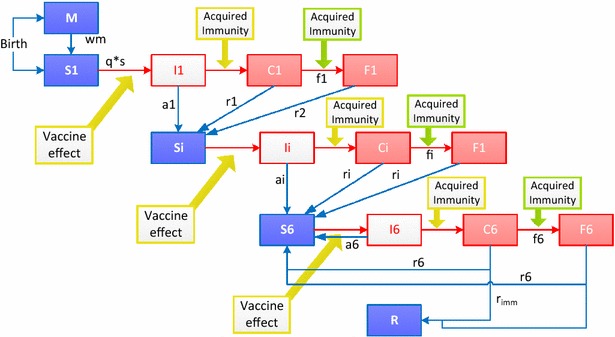
Model structure. *M* maternal protection, *S* susceptible, *I* infected (parasites emerging from the liver), *C* clinical disease episode, *F* severe disease episode and there are 6 levels of immunity with compartments *S*, *I*, *C* and *F* divided into 6 levels, *R* resistant, *wm* waning of maternal immunity, *q* probability of infection, *s* susceptibility to infection as a function of age, *a* probability of asymptomatic infection, *r* recovery rate from clinical disease, *w* waning rate of acquired immunity, *r*_*imm*_ probability of developing full immunity. The model assumes two processes for acquisition of immunity, one process that protects against clinical malaria of any severity and a faster process that protects against severe malaria

The model is a static Markov cohort model with an individual-based stochastic process following a birth cohort over 10 years. It includes three categories of malaria transmission intensity, using definitions consistent with the Malaria Atlas Project (MAP) [11]. Low intensity is defined as parasite prevalence (PP) ≤5 %, moderate intensity as PP 5–40 %, and high intensity as PP > 40 % in children 2–10 years old. The structure of the model is shown in Fig. 1. At birth, individuals are either protected by maternal antibodies (state M) or susceptible (state S). Newborns protected by maternal antibodies gradually lose that immunity at a fixed rate (wm) to become susceptible. From a susceptible state they may be infected with malaria (state I). The transition probability for moving from state S to state I is the product of two parameters, a fixed probability of infection for each transmission category (q) and an age-dependent susceptibility factor (s). Infected individuals (state I) may have an asymptomatic episode with a probability (a) which depends on their immunity level or they may develop clinical symptoms (state C). A clinical episode may either be uncomplicated and be resolved, in which case individuals return to the susceptible state (state S) with enhanced immunity, or lead to a severe episode (state F) in a fixed proportion which also depend on the level of immunity. A fixed proportion of severe episodes (state F) lead to death. The death state is not represented in the Figure, but it is included in the model. Six successive immunity levels after each infection (designated S_1_–S_6_, etc., in the Figure) are included in the model. This means that for a higher level of immunity the probability for a new infection to cause a clinical episode and for a clinical episode to become severe are lower than for the previous stage of immunity. The number of immunity levels is limited but children may experience a larger number of infections. These additional infections would not improve natural immunity any more but for each infection a fixed proportion would result in a fully resistant state (state R). Although there is no sterilizing immunity to parasites, this model structure accounts for this resistant stage with full immunity against clinical episodes. This resistant state might be only a transient one, therefore a waning of naturally acquired immunity is tested with additional transitions from R to S_6_ and S_i+1_ to S_i_ included in the calibration process.

Key assumptions include:

- All episodes of clinical and severe malaria occurring in the trial were reported;
- There are six levels of acquired immunity to clinical malaria depending on the previous number of infections;
- A fixed percentage of severe infections result in death;
- The effect of the vaccine is only to reduce the risk of infection, with no additional effect on the risk of developing clinical or severe disease;
- Vaccine efficacy wanes over time by exponential decay;
- No seasonality is included. Only one site (Nanoro in Burkina Faso) of the 11 trial sites exhibits a strongly seasonal pattern [10] which limits the availability of data to calibrate the model.

### Incidence data

Each site in the Phase III trial was assigned to a transmission category using PP data from the Malaria Transmission Intensity (MTI) study, which was conducted at the same sites as the Phase III trial but in different children [12]. The incidence of clinical malaria in the control group at sites in each transmission category was obtained from the Phase III trial statistical report, and 95 % Chi-square CI were derived using the method described by Ulm [13]. The secondary case definition 1 (malaria episode defined as a child presenting with fever >37.5 °C and with *P. falciparum* parasites in the blood) was used because of the better sensitivity of this measure of malaria burden. The data are shown in Table 1.

**Table 1:** Incidence of clinical and severe malaria in the control group of the Phase III trial

	Low (PP ≤ 5 %)	Moderate (PP 5–40 %)	High (PP > 40 %)	Overall
Sites	Kilifi (Kenya), Korogwe (Tanzania), Manhiça (Mozambique)	Bagamoyo (Tanzania), Lambarene (Gabon), Agogo (Ghana), Kintampo (Ghana), Lilongwe (Malawi)	Siaya (Kenya), Kombewa (Kenya), Nanoro (Burkina Faso)	
Control group incidence (95 % CI) of clinical malaria per child per year in each 6–month follow-up period for infants receiving the first vaccine dose at age 6–12 weeks				
0–6 months	na	0.74 (0.65–0.83)	2.82 (2.62–3.03)	1.20 (1.13–1.28)
6–12 months	0.13 (0.088–0.185)	0.96 (0.87–1.06)	3.69 (3.46–3.93)	1.54 (1.46–1.62)
12–18 months	0.20 (0.14–0.27)	0.91 (0.82–1.01)	3.35 (3.13–3.59)	1.50 (1.42–1.59)
Control group incidence (95 % CI) of clinical malaria per child per year in each 6–month follow-up period for children receiving the first vaccine dose at age 5–17 months				
0–6 months	0.058 (0.030–0.101)	1.10 (1.00–1.19)	3.32 (3.12–3.53)	1.56 (1.48–1.64)
6–12 months	0.13 (0.09–0.19)	1.49 (1.39–1.60)	4.59 (4.35–4.83)	2.11 (2.02–2.20)
12–18 months	na	1.14 (1.04–1.24)	3.46 (3.25–3.67)	1.67 (1.59–1.75)
Incidence of severe malaria per 1000 children (95 % CI) per year across the whole follow-up period				
Infants receiving the first vaccine dose at age 6–12 weeks	2.51 (0.06–14.10)	27.2 (17.0–41.1)	56.3 (41.7–74.5)	39.0 (27.7–38.4)
Children receiving the first vaccine dose at age 5–17 months	4.62 (0.95–13.5)	39.0 (29.8–50.2)	61.2 (47.1–78.2)	39.3 (32.7–46.7)

Clinical malaria defined as illness accompanied by a temperature of at least 37.5 °C or reported fever in the last 24 h and asexual *P. falciparum* parasites in the blood at a density of >0 parasites/mm^3^

Severe malaria defined as *P. falciparum* parasites in the blood at a density of >5000 parasites/mm^3^

*na* not available, *PP* parasite prevalence

### Model calibration

The model was calibrated to reproduce simultaneously the incidence of malaria observed in the control arm of the Phase III trial for both age groups (infants and children) in each transmission category and each six-month follow-up period, and the age distribution up to the age of 5 years in moderate and high transmission areas with no marked seasonality reported by Carneiro et al. [14]. Since different definitions of transmission intensity categories were used, high transmission intensity data from the model were compared against moderate transmission intensity data from Carneiro et al., and moderate transmission data from the model were compared against an average calculated from moderate and low transmission data from Carneiro et al. The age distribution in low transmission areas was not included because the definition of low transmission used by Carneiro et al. (PP ≤ 25 %) [14] was too different from the definition used in the model (PP ≤ 5 %).

This calibration of incidence to the observed data from the control arm of the trial was performed by fitting the following parameters (see Fig. 1):

- Infection probability for each transmission category (q);
- Probability of asymptomatic infection with each successive infection (a1–a6);
- Susceptibility factor (s) representing an increase in susceptibility with age to account for higher probability of mosquito bites with larger body surface, is derived from a negative exponential equation of the following type: 1 − exp(−k*n), where ‘n’ is the age of the child expressed as the number of model cycles (5 days per cycle) and ‘k’ is derived by calibration;
- Waning of immunity (w) implemented as a transition from S_i+1_ to S_i_;
- Percentage of individuals gaining full immunity after six infections (r_imm_).

Calibration was performed by the least square method using Matlab (version R2013b) minimization tools (interior-point algorithm). To ensure that immunity always increased with successive infections, a constraint was set so that ai was always less than or equal to ai+1 for values of i from 1 to 5.

### Capturing uncertainty and heterogeneity

A main source of uncertainty comes from the observation of incidence rates and vaccine efficacy estimates of the Phase III trial. Although the Phase III trial is powered for clinical malaria incidence and severe malaria incidence overall, larger variations appear when stratifying data by malaria transmission level and periods of follow-up. In order to capture this uncertainty, model parameter distributions are generated by repeating the calibration process with several incidence rates. These incidence rates are randomly drawn from a log-normal distribution generated using median values and 95 % CI for each 6-month follow-up period and for each age-group.

This produced a set of values for the calibrated parameters from which non-parametric distributions were derived. A random value is drawn from the corresponding non-parametric distribution for each parameter to simulate the model process for one individual. Table 2 shows the point estimates and CIs for the parameters. Additional file 1: Figure S1 shows the distributions for q parameters and Additional file 2: Figure S2 shows the distributions for parameters a1 to a6 and f1 to f6.

**Table 2:** Point estimates, 95 % confidence intervals and distributions of fitted parameters

Parameter	Point estimate	95 % CI	Distribution
q, low transmission	1.88 e−3	1.224 e−3, 2.217 e−3	Non-parametric
q, moderate transmission	30.50 e−3	19.222 e−3, 37.682 e−3	Non-parametric
q, high transmission	184.8 e−3	87.972 e−3, 272.147 e−3	Non-parametric
Probability of full immunity, *r*_imm_	1.97 %	1.93 %, 2.17 %	Non-parametric
Age-related susceptibility factor, k	1.58 e−2	1.02 e−2, 3.63 e−2	Non-parametric
Waning of acquired immunity, w	0		
Probability of asymptomatic infection			
a1	8.57 %	0.29, 17.79 %	Non-parametric
a2	38.49 %	7.74, 43.62 %	Non-parametric
a3	38.58 %	7.94, 43.82 %	Non-parametric
a4	38.68 %	8.37, 43.93 %	Non-parametric
a5	38.90 %	15.65, 44.12 %	Non-parametric
a6	54.19 %	25.87, 61.82 %	Non-parametric
Percentage of clinical cases that become severe with good access to care			
f1	2.40 %	1.19, 5,46 %	Non-parametric
f2	2.13 %	1.19, 3.04 %	Non-parametric
f3	2.13 %	1.19, 3.03 %	Non-parametric
f4	2.05 %	1.19, 2.92 %	Non-parametric
f5	1.99 %	1.19, 2.92 %	Non-parametric
f6	1.33 %	0.96, 2.12 %	Non-parametric
Vaccine parameters			
Vaccine efficacy in infants aged 6–12 weeks	37.6 %	35.53, 41.11 %	Log-normal
Half-life in infants aged 6–12 weeks (months)	6.2	3.3, 11.7	Log-normal
Vaccine efficacy in children aged 5–17 months	58.2 %	56.5, 59.6 %	Log-normal
Half-life in children aged 5–17 months (months)	14.4	10.6, 19.2	Log-normal

The distribution of the number malaria episodes per child in the Phase III trial in each transmission category indicated an individual-level heterogeneity in the risk of infection, as the proportion of children with no malaria episodes was higher in moderate and high transmission sites than would be expected with a homogeneous risk of infection. This heterogeneity in exposure was captured by assuming an individual-level variation around a mean exposure level corresponding to each of the three transmission levels included in the model.

### Severe malaria cases and hospitalizations

Data on the incidence of severe cases (defined as a parasite density of >5000) per 1000 person-years for each transmission category were obtained from the Phase III trial [10] (Table 1). A calibration step fitted the model parameters for the percentage of clinical cases becoming severe at each of the six modelled successive infections (f1–f6) to the incidence of severe cases observed in the Phase III trial. A constraint of diminishing percentages of clinical cases becoming severe with increasing number of previous infections (f_i+1_ < f_i_) is imposed to reflect the acquisition of immunity against severe malaria. This constraint results in a faster acquisition of immunity against severe disease than immunity against clinical disease.

The ratio of severe malaria cases to hospitalized malaria cases (defined as a parasite density of >5000 and excluding planned admissions and trauma) was calculated for each transmission category from the Phase III trial. However, the level of care available to patients in the Phase III trial was very high, with all malaria cases having access to high levels of treatment resulting in very low malaria mortality. This is not representative of the real-life situation in sub-Saharan Africa, where not all malaria cases will have access to prompt treatment and/or hospital care. Thus, compared with real-world situations, the Phase III trial would be expected to have a higher percentage of severe cases receiving hospital care, and a lower percentage of cases that become severe. The model thus required adjustment to reflect limited access to treatment for uncomplicated cases, and limited access to hospitalization for severe cases, as could be expected in real-life settings.

The proportion of cases in real-life settings with access to artemisinin-based combination treatment was taken from a published study for Ghana, Kenya and Tanzania [15], with the average of these countries (fixed value of 54 %) used for other countries. For the country-level estimates, the percentage of severe cases that were hospitalized in real-life settings was assumed to be the same as the percentage with access to artemisinin-based combination treatment [15].

The risk of developing a severe episode from a treated uncomplicated episode was calibrated from the trial data as described above. The relative risk of developing a severe episode from an untreated uncomplicated episode was calculated using the following formula:

Severe malaria in population = (Proportion untreated × relative risk + proportion treated) × severe malaria in model

Proportion treated = Proportion of cases promptly treated with artemisinin-based combination therapy in various countries [16], and see also the associated online supplementary materials [17];

Severe malaria in model = Estimated incidence of severe malaria in children aged <5 years predicted by model;

Severe malaria in population = Incidence of severe malaria in hospitals in children aged 0–9 years [18], corrected for the proportion of severe episodes in children aged <5 years using the age distribution from Carneiro et al. [14] and for the proportion of severe cases who are not hospitalized [19].

This calculation was carried out for a range of countries in sub-Saharan Africa, and the mean relative risk across all the countries was 1.84 (95 % CI 1.68, 2.01). Table 3 shows the values used for calculating severe malaria and hospitalizations.

**Table 3:** Values for fixed parameters

Parameter	Value	Source
Vaccine efficacy after 1 dose	None	Assumption
Vaccine efficacy after 2 doses	One quarter of the vaccine efficacy of a full course (see Table 2)	Assumption
Maternal protection, % of infants protected	95 %	Assumption
Waning of maternal protection, wm	3 months half-life	Similar to previous models
Probability of recovery from clinical disease, r	1/3 Half recover after 15 days	Assumption for a context of good access to care
Access to ACT treatment	Ghana: 55 %, Kenya: 45 %, Tanzania: 62 % and country surveys for 22 countries Average of 31 % for the remaining 17 countries	Sicuri [15] and Demographic and Health Surveys and Malaria Indicators Surveys
% of severe cases hospitalized	54 %	Assumed same as access to ACT in public health facilities in Ghana, Kenya and Tanzania
Relative risk for an untreated uncomplicated episode becoming severe (compared with a treated uncomplicated episode)	1.84 (95 % CI 1.68, 2.01)	Calculated from the modelled number of severe cases, the % of severe cases hospitalized, and access to treatment [16]
% of severe malaria cases resulting in sequelae	1.7 % (range 0.85–2.54 %)	Calculated from the % of cerebral malaria cases with sequelae from WHO report [21], and the % of severe cases that are cerebral malaria [20]
Case-fatality rate, CFR (% of severe cases)		
Treated	13.6 % (95 % CI 8.4, 18.8 %)	Thwing [22]
Untreated	3 × treated cases 40.8 % (95 % CI 25.2, 56.4 %)	Thwing [22]

*ACT* artemisinin-based combination therapy, *CI* confidence interval

### Long-term sequelae and deaths

The proportion of severe cases with long-term sequelae was calculated from published data on the proportion of severe cases that are categorized as cerebral malaria [20] and the percentage of cerebral malaria cases with sequelae [21]. The mean age of children presenting with cerebral malaria is about 3 years compared to 1.8 for those presenting with malarial anaemia [21]. Malaria mortality was estimated as a fixed percentage of severe cases (case-fatality rate), with values for hospitalized and non-hospitalized severe cases obtained from Thwing et al. [22] (Table 3).

### Calibration of vaccine effect

The vaccine effect was modelled by applying a reduction in the force of infection (i.e., a reduction in the probability of moving from state S to state I in each successive infection). This type of vaccine effect was considered in order to reproduce the mechanism of action of RTS,S targeting the parasite at the pre-erythrocytic stage. It was assumed that vaccine efficacy would wane over time following an exponential decay function.

Point estimates and 95 % CI for vaccine efficacy were calculated from the Phase III clinical trial data for each of the three transmission categories and each of the two vaccination age groups using the exact Fisher formula [23].

The point estimates for the initial reduction in the force of infection post-dose 3 (or vaccine efficacy against infection) and decay parameter in the model were fitted to the vaccine efficacy observed in the Phase III trial simultaneously for each transmission level, each six-month follow-up period and the entire 18-month follow-up period. The upper and lower bounds of the 95 % CI for vaccine efficacy against infection were obtained by fitting simultaneously the upper and lower values for each six-month follow-up period and the 18-month follow-up period. For the vaccine waning parameter, the upper (lower) bound was derived by combining the upper (lower) bound, point estimates and lower (upper) bound of vaccine efficacy from the three successive six-month periods and the 18-month point estimate for vaccine efficacy. The fitted parameters for vaccine efficacy and the half-life of the waning function for each vaccination age group are shown in Table 2. Vaccine efficacy rates after one or two doses were set at fixed values and are shown in Table 3.

### Estimation of country-level impact

Estimates of the malaria burden in the absence of vaccination and the potential impact of vaccination were made for 42 countries in sub-Saharan Africa. For each country, the size of the annual birth cohort and all-cause child mortality rates were taken from United Nations data [24, 25]. The birth cohort was then allocated between the three transmission intensity categories using 2010 MAP data for that country [11]. In some countries a fraction of the birth cohort was considered not at risk or with an unstable risk of malaria. No impact of vaccination was considered in that population. The percentage of cases with access to treatment was taken from a published survey [15] for Ghana, Kenya and Tanzania and Demographic and Health and Malaria Surveys in 22 additional countries representing about 80 % of the birth cohorts of the 42 countries. The average of 31 % access to anti-malarial treatment from the 25 countries in these surveys was used for remaining countries. The percentage of severe cases hospitalized and the case-fatality rate were set at fixed values for all countries (see Table 3). Running the model with these input data produced estimates of the numbers of clinical malaria cases, severe malaria cases, malaria hospitalizations and malaria deaths for each country. The proportion of the population in each malaria transmission category was assumed to remain stable over the projected period of 10 years. Data are presented in this paper for all 42 countries combined and for 8 individual countries: two countries with a large malaria burden (Democratic Republic of Congo and Nigeria accounting for 39 % of global malaria cases and 34 % of global malaria deaths [1]) and four of the countries in the Phase III trial (Burkina Faso, Ghana, Kenya, and Tanzania) and two additional countries (Senegal and Uganda). Details for each country are presented in Additional file 3: Table S1.

### Simulation process

The model cycle time was 5 days. For each transmission level, 50 stochastic simulations of a cohort of 10,000 individuals were conducted. For each simulation, parameter values for each of the 10,000 individuals in the cohort were randomly drawn from the distributions shown in Table 2. For the parameter q, which is the fixed probability of infection for each transmission category, there was also a second step to account for individual heterogeneity in exposure. After the value for q in the relevant transmission category (low, moderate or high) was drawn from the distribution, it was further adjusted for each individual in the cohort by a factor drawn from a uniform distribution ranging from 0 to 2 q. The model then followed the cohort from birth to the age of 10 years, with one of the following interventions:

- Without malaria vaccination;
- With RTS,S malaria vaccination with doses given at age 6, 10 and 14 weeks (corresponding to the age group vaccinated at age 6–12 weeks in the trial, and to addition of the malaria vaccine to the Expanded Programme on Immunization (EPI) schedule), with coverage assumed to be the same as the diphtheria-tetanus-pertussis (DTP3) vaccination coverage from each country;
- With RTS,S malaria vaccination with doses given at age 6, 7-and-a-half and 9 months (corresponding to the age group vaccinated at age 5–17 months in the trial).

The vaccine efficacy in the 5–17 months age groups was used for children receiving the first dose at 6 months of age. Efficacy did not change between children receiving the first dose before or after 12 months of age. The coverage was assumed to reach 75 % of DTP3 vaccination coverage from each country to account for the difficulty to reach children at a later age compared to DTP schedule. The modelled outcomes were the numbers of clinical malaria cases, severe malaria cases, malaria hospitalizations and malaria deaths expected in the cohort from birth to the age of 10 years. This process was repeated 50 times with and without vaccination and the values compared to estimate the number of events averted by vaccination for each outcome. At each year, the median value and the 2.5th and 97.5th percentile values (corresponding to the 95 % CI) were recorded for each event averted.

### Sensitivity analysis

Univariate sensitivity analysis was conducted by running the model with different values for transmission, access to treatment, duration of vaccine protection, vaccine coverage, risk of mortality in the community, relative risk of severe malaria for untreated cases, and case-fatality rate. The other parameters are held constant with median values provided in Tables 2 and 3.

An additional scenario analysis was conducted to compare the model with estimates from the WHO World Malaria Report 2012 [26].

## Results

### Model calibration and validation

**Fig. 2:**
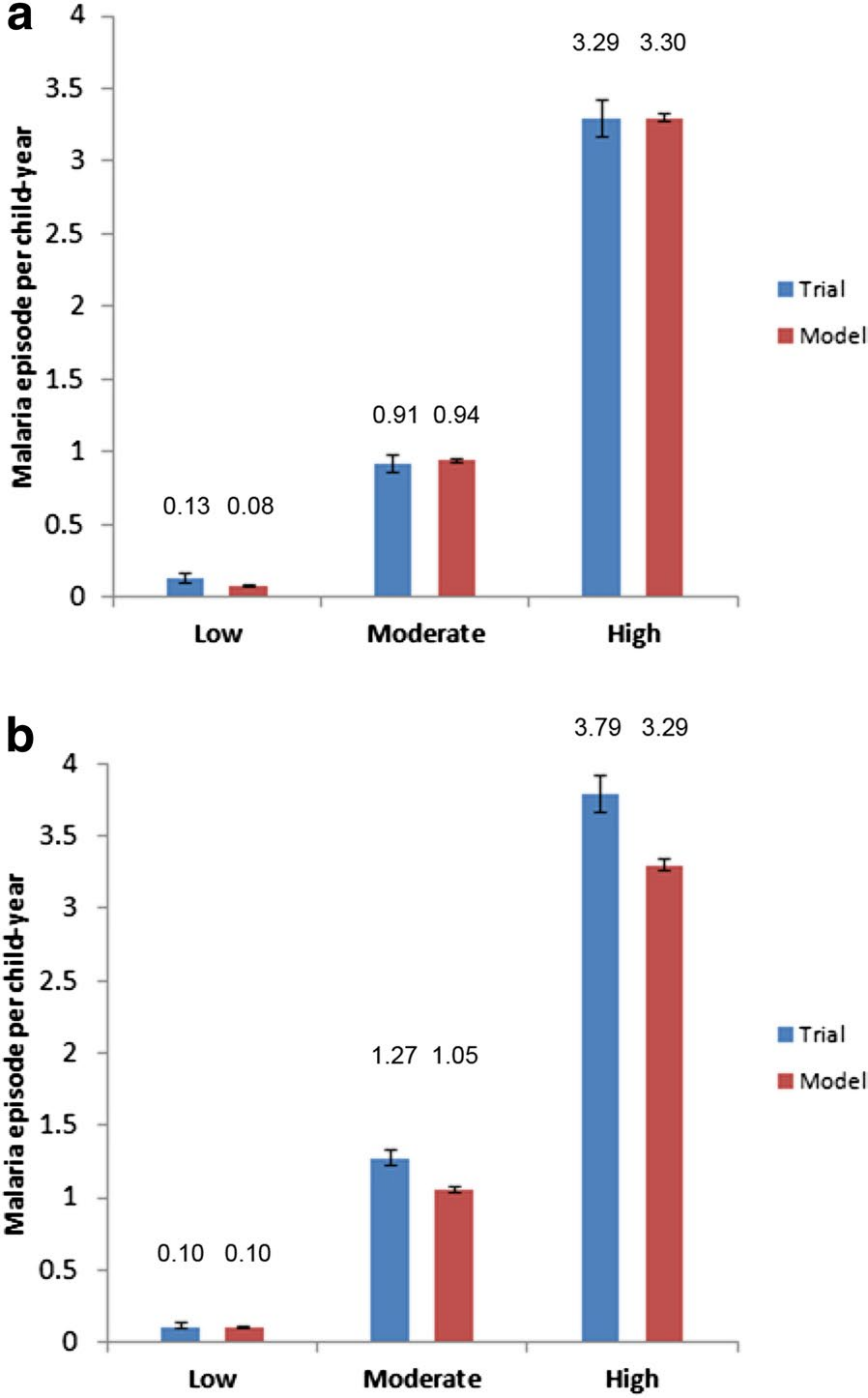
Model validation, incidence of clinical malaria. Incidence of clinical malaria (defined as in Table 1) per child per year over the 18-month follow-up period observed in the control arm of the Phase III trial and predicted by the model in each transmission category in **a** the 6–12 weeks age group and **b** the 5–17 months age group

Figure 2 shows the incidence of clinical malaria over the 18-month follow-up period observed in the control arm of the Phase III trial and estimated by the model, for each age group and each transmission level. The modelled results matched the trial results closely, although some differences appeared mainly in the infant age group for the low transmission category and in the older age group for moderate and high transmission. The model mostly underestimates the malaria incidence observed in the trial for the 5 to 17-month group.

Table 4 shows the number of clinical malaria cases and the percentage of severe malaria cases observed in the control and vaccine arms of the Phase III trial and predicted by the model. The modelled results matched well with the data (error in acceptable 10 % area) in the age group vaccinated at age 6–12 weeks although in the vaccine arm the model provided more severe cases than the trial (+23 %). In the age group vaccinated at 5–17 months the data were more difficult to reproduce because of the lower number of cases estimated in moderate and high transmission settings. The model tended to underestimate the burden of malaria compared with the observed data from the trial in the 5–17 months as pointed out in Table 4.

**Table 4:** Model validation, clinical and severe malaria in vaccine and control arms

	Trial	Model	% Delta
Age group 6–12 weeks			
Clinical episodes			
Control	3718	3798	+2.2
Vaccine	4487	4802	+7.0
Severe episodes (% of clinical episodes)			
Control (%)	2.76	2.70	−2.2
Vaccine (%)	2.91	3.58	+23
Age group 5–17 months			
Clinical episodes			
Control	5409	4967	−8.2
Vaccine	5133	4154	−19
Severe episodes (% of clinical episodes)			
Control (%)	2.20	1.96	−10.9
Vaccine (%)	2.27	2.69	+18.5

Number of cases of clinical malaria (defined as in Table 1) over the 18-month follow-up period observed in the control and vaccine arms of the Phase III trial and predicted by the model in each age group

Twice as many patients were randomized to the vaccine arm as the control arm

**Fig. 3:**
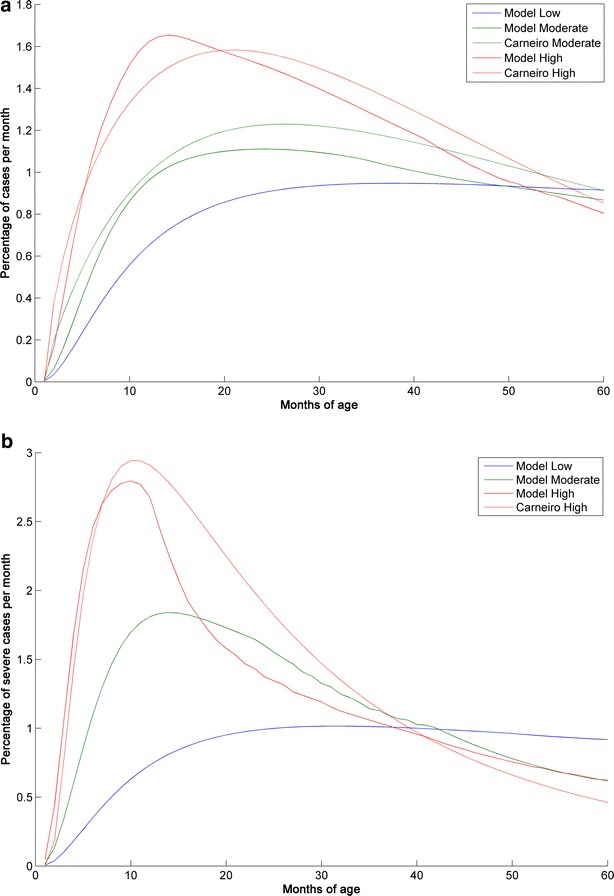
Age distribution of clinical and severe malaria cases predicted by the model compared with published data. Comparison of the age distribution of **a** clinical malaria cases and **b** severe malaria cases predicted by the model with age distribution data published by Carneiro et al. [14]. To allow for differences in transmission intensity definitions, the high transmission data from the model were compared with the moderate transmission data from Carneiro et al., and for clinical cases the moderate transmission data from the model were compared against an average calculated from the moderate and low transmission data from Carneiro et al. Severe cases were compared against the hospitalized cases from Carneiro et al. for which data are not available for low transmission settings without seasonality. The Y axis shows the percentage of events occurring in a particular month of age out of the total number of events during the observation period

The age distribution of malaria cases predicted by the model matched well with the age distribution published by Carneiro et al. [14] for clinical cases (Fig. 3a) and severe cases (Fig. 3b).

### Estimated public health impact of adding RTS,S vaccination to existing interventions

**Fig. 4:**
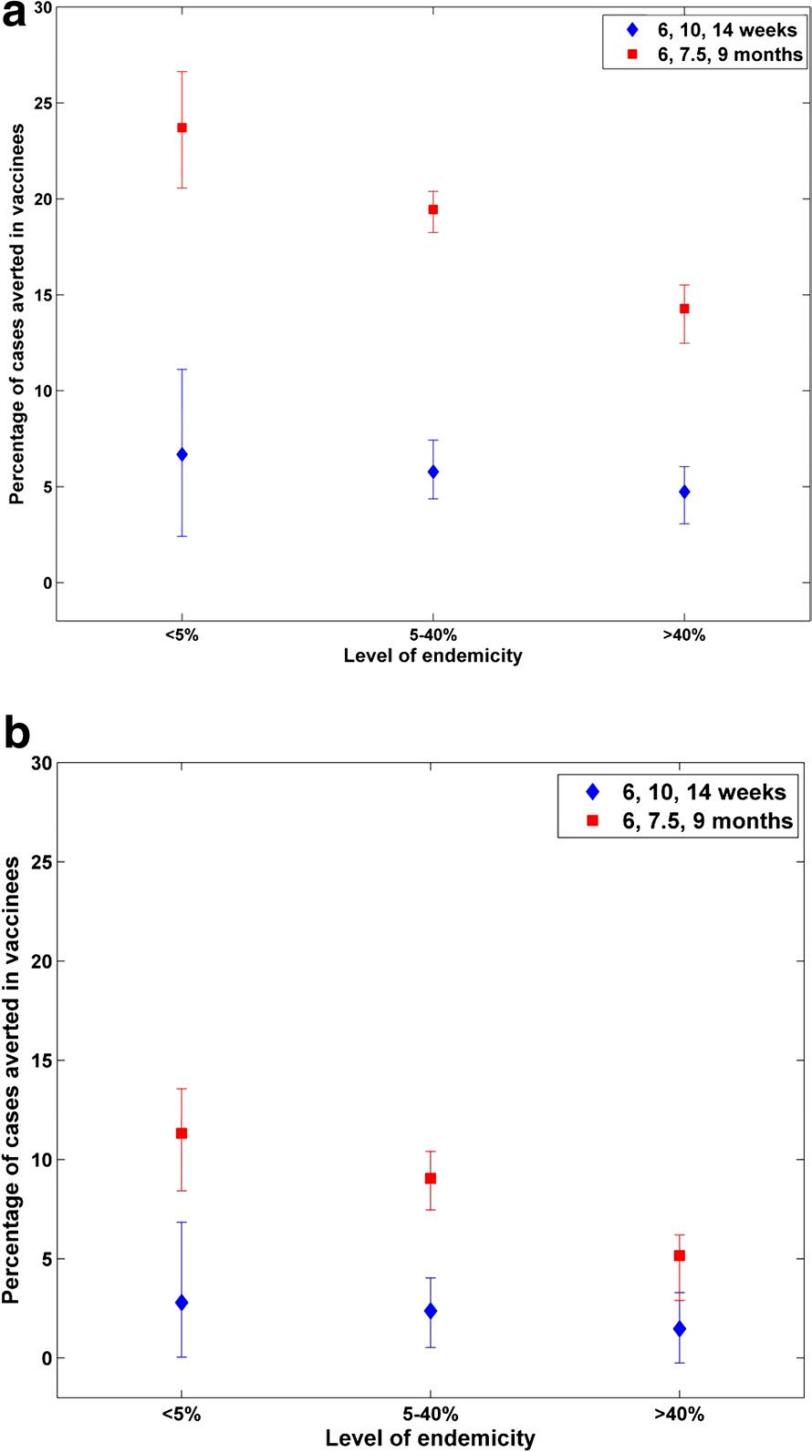
Predicted percentage of clinical malaria cases averted with RTS,S vaccination at age 6, 10 and 14 weeks and at age 6, 7-and-a-half and 9 months in children under 5 years of age (**a**) and in children under 10 years of age (**b**) for low (PP < 5 %), moderate (5 < PP < 40 %) and high transmission levels (PP > 40 %)

Figure 4 shows the estimated percentage of clinical malaria cases averted when adding RTS,S vaccination at age 6, 10 and 14 weeks and at age 6, 7-and-a-half and 9 months in low, moderate and high transmission categories. Addition of RTS,S would be expected to reduce the incidence of clinical malaria in a higher proportion for low transmission than higher transmission settings. The benefit would be expected to occur in the first 1–4 years, and the model predicted a small increase in later years in high transmission areas, reflecting delayed development of natural immunity. This appears in the reduction of proportion of events averted in children less than 10 years of age compared with children under 5 years of age.

### Estimated country-level public health impact of adding RTS,S vaccination

Table 5 shows the estimated impact of adding RTS,S vaccination with doses administered at age 6, 10 and 14 weeks or 6, 7-and-a-half and 9 months in 42 African countries and eight specific countries (see Additional file 3: Table S1 for results in each country). The estimates were based on vaccinating the birth cohort in 2017 and following them for 10 years, assuming no change in malaria transmission. Vaccination at age 6, 10 and 14 weeks would be expected to avert almost five million cases of clinical malaria, 119,000 severe malaria cases, 98,600 malaria hospitalizations and 31,000 malaria deaths. Vaccination at age 6, 7-and-a-half and 9 months would be expected to avert almost 12.5 million cases of clinical malaria, 250,400 severe malaria cases, 208,000 malaria hospitalizations and 65,400 malaria deaths. Therefore the higher efficacy obtained in five to 17-months age group would overcome the lower coverage with vaccination starting at 6 months (75 % of DTP3).

**Table 5:** Estimated country-level impact of RTS,S vaccination

Country	Number of vaccinees	Median (95 % CI)			
		Clinical malaria cases averted	Severe malaria cases averted	Malaria hospitalizations averted	Malaria deaths averted
Vaccination at 6, 10 and 14 weeks					
Burkina Faso	673,961	234,951 (2090, 477,517)	4182 (−6846, 14,515)	3474 (−5688, 12,059)	1092 (−1788, 3,790)
Democratic Republic of Congo	2,177,255	590,733 (77,960, 1,114,625)	13, 943 (−22,087, 41,080)	11, 583 (−18,349, 34,128)	3641 (−5767, 10,727)
Ghana	739,857	180,785 (17,756, 344,188)	4080 (−8121, 11,908)	3389 (−6747, 9893)	1065 (−2121, 3109)
Kenya	1,423,439	96,415 (12,032, 189,548)	2805 (−6042, 9841)	2330 (−5020, 8176)	732 (−1578, 2570)
Nigeria	3,641,124	1,137,914 (106,434, 2,194,118)	25,223 (−41,239, 77,330)	20,955 (−34,261, 64,243)	6586 (−10,768, 20,192)
Tanzania	1,879,173	255,645 (37,085, 478,220)	6484 (−13,984, 19,374)	5387 (−11,618, 16,096)	1693 (−3,652, 5059)
Senegal	469,921	36,048 (7275, 67,343)	1477 (−3337, 4803)	1227 (−2772, 3990)	386 (−871, 1254)
Uganda	1,462,572	411,047 (11,695, 819,896)	7480 (−14,269, 24,160)	6214 (−11,854, 20,071)	1953 (−3726, 6309)
42 countries in sub-Saharan Africa	25,210,481	5,004,489 (474,867, 9,684,160)	118,736 (−214,795, 372,995)	98,642 (−178,445, 309,873)	31,004 (−56,087, 97,396)
Vaccination at 6, 7.5 and 9 months					
Burkina Faso	488,861	562,795 (327,910, 669,793)	9985 (−54, 19,791)	8295 (−45, 16,441)	2607 (−14, 5168)
Democratic Republic of Congo	1,551,474	1,414,342 (923,277, 1,657,736)	28,922 (1270, 54,286)	24,028 (1,055, 45,099)	7552 (332, 14,175)
Ghana	541,400	468,774 (323,528, 549,868)	8254 (82, 15,470)	6857 (69, 12,852)	2155 (22, 4040)
Kenya	1,041,584	260,446 (189,489, 307,495)	5,641 (−636, 11,453)	4,686 (−528, 9,515)	1473 (−166, 2991)
Nigeria	2,624,558	2,749,346 (1,748,642, 3,234,411)	54,546 (1758, 103,606)	45,315 (1460, 86,073)	14,243 (459, 27,054)
Tanzania	1,382,820	693,506 (515,124, 811,055)	12,453 (−451, 23,670)	10,346 (−375, 19,665)	3252 (−118, 6181)
Senegal	344,479	101,544 (80,459, 118,862)	2741 (−315, 5441)	2277 (−262, 4520)	716 (−82, 1421)
Uganda	1,072,051	1,038,791 (657,896, 1,229,031)	16,671 (−72, 32,293)	13,850 (−60, 26,828)	4353 (−19, 8432)
42 countries in sub-Saharan Africa	18,313,808	12,484,309 (8,242,412, 14,696,728)	250,448 (−433, 481,819)	208,064 (−359, 400,280)	65,397 (−113, 125,813)

Estimated numbers of vaccinees and median and 95 % CIs for the number of clinical malaria cases, severe malaria cases, malaria hospitalizations and malaria deaths averted by RTS,S vaccination with doses administered either at 6, 10 and 14 weeks or at 6, 7-and-a-half and 9 months

Outcomes are estimated for the annual birth cohort in each country, beginning in 2017 and following the cohort until the age of 10 years, assuming no change in malaria transmission

Table 6 shows these estimated impact data expressed per 100,000 vaccinees. Countries with high malaria transmission, such as Nigeria and Burkina Faso, generally had higher values than countries with lower malaria transmission, such as Kenya. In Kenya more than 75 % of the population is not exposed or exposed to low levels of malaria transmission (i.e., with parasite prevalence below 5 % in children of 2–10 years old) while 7 % would experience high malaria transmission (i.e., with parasite prevalence above 40 % in children of 2–10 years old). Exposure levels are also variable in Tanzania with about 45 % of the population in low transmission areas versus 12 % experiencing high levels of transmission. This suggests that the projected impact of vaccination increases with transmission even if the relative percentage of events averted is predicted to decrease with transmission. Vaccination at age 6, 7-and-a-half and 9 months generally resulted in higher values than vaccination at age 6, 10 and 14 weeks, reflecting the higher vaccine efficacy in the older age group.

**Table 6:** Estimated country-level impact of RTS,S vaccination per 100,000 vaccinees

Country	Median (95 % CI)			
	Clinical malaria cases averted	Severe malaria cases averted	Malaria hospitalizations averted	Malaria deaths averted
Vaccination at 6, 10 and 14 weeks				
Burkina Faso	34,861 (310, 70,852)	621 (−1016, 2154)	516 (−844, 1789)	162 (−265, 562)
Democratic Republic of Congo	27,132 (3581, 51,194)	640 (−1014, 1887)	532 (−843, 1567)	167 (−265, 493)
Ghana	24,435 (2400, 46,521)	551 (−1098, 1610)	458 (−912, 1337)	144 (−287, 420)
Kenya	6773 (845, 13,316)	197 (−424, 691)	164 (−353, 574)	51 (−111, 181)
Nigeria	31,252 (2923, 60,259)	693 (−1133, 2124)	576 (−941, 1764)	181 (−296, 555)
Tanzania	13,604 (1973, 25,448)	345 (−744, 1031)	287 (−618, 857)	90 (−194, 269)
Senegal	7671 (1548, 14,331)	314 (−710, 1022)	261 (−590, 849)	82 (−185, 267)
Uganda	28,104 (800, 56,058)	511 (−976, 1652)	425 (−811, 1372)	134 (−255, 431)
42 countries in sub-Saharan Africa	19,851 (1884, 38,413)	471 (−852, 1480)	391 (−708, 1229)	123 (−222, 386)
Vaccination at 6, 7.5 and 9 months				
Burkina Faso	115,124 (67,076, 137,011)	2042 (−11, 4048)	1697 (−9, 3363)	533 (−3, 1057)
Democratic Republic of Congo	91,161 (59,510, 106,849)	1864 (82, 3499)	1549 (68, 2907)	487 (21, 914)
Ghana	86,586 (59,758, 101,564)	1524 (15, 2857)	1267 (13, 2374)	398 (4, 746)
Kenya	25,005 (18,192, 29,522)	542 (−61, 1100)	450 (−51, 914)	141 (−16, 287)
Nigeria	104,755 (66,626, 123,236)	2078 (67, 3948)	1727 (56, 3280)	543 (17, 1031)
Tanzania	50,152 (37,252, 58,652)	901 (−33, 1712)	748 (−27, 1422)	235 (−9, 447)
Senegal	29,478 (23,357, 34,505)	796 (−92, 1580)	661 (−76, 1312)	208 (−24, 412)
Uganda	96,898 (61,368, 114,643)	1555 (−7, 3012)	1292 (−6, 2502)	406 (−2, 787)
42 countries in sub-Saharan Africa	68,169 (45,007, 80,249)	1368 (−2, 2631)	1136 (−2, 2186)	357 (−1, 687)

Estimated median and 95 % CIs for the numbers of clinical malaria cases, severe malaria cases, malaria hospitalizations and malaria deaths per 100,000 vaccinees averted by RTS,S vaccination with doses administered either at 6, 10 and 14 weeks or at 6, 7-and-a-half and 9 months

Outcomes are estimated for the annual birth cohort in each country, beginning in 2017 and following the cohort until the age of 10 years, assuming no change in malaria transmission

Over the 42 African countries it is estimated that 2019 (−3652; 6341) cases of neurological sequelae would be averted with vaccination at the age of 6, 10 and 14 weeks and 4258 (−7; 8191) cases of neurological sequelae with vaccination at the age of 6, 7-and-a-half and 9 months.

### Sensitivity analysis

**Fig. 5:**
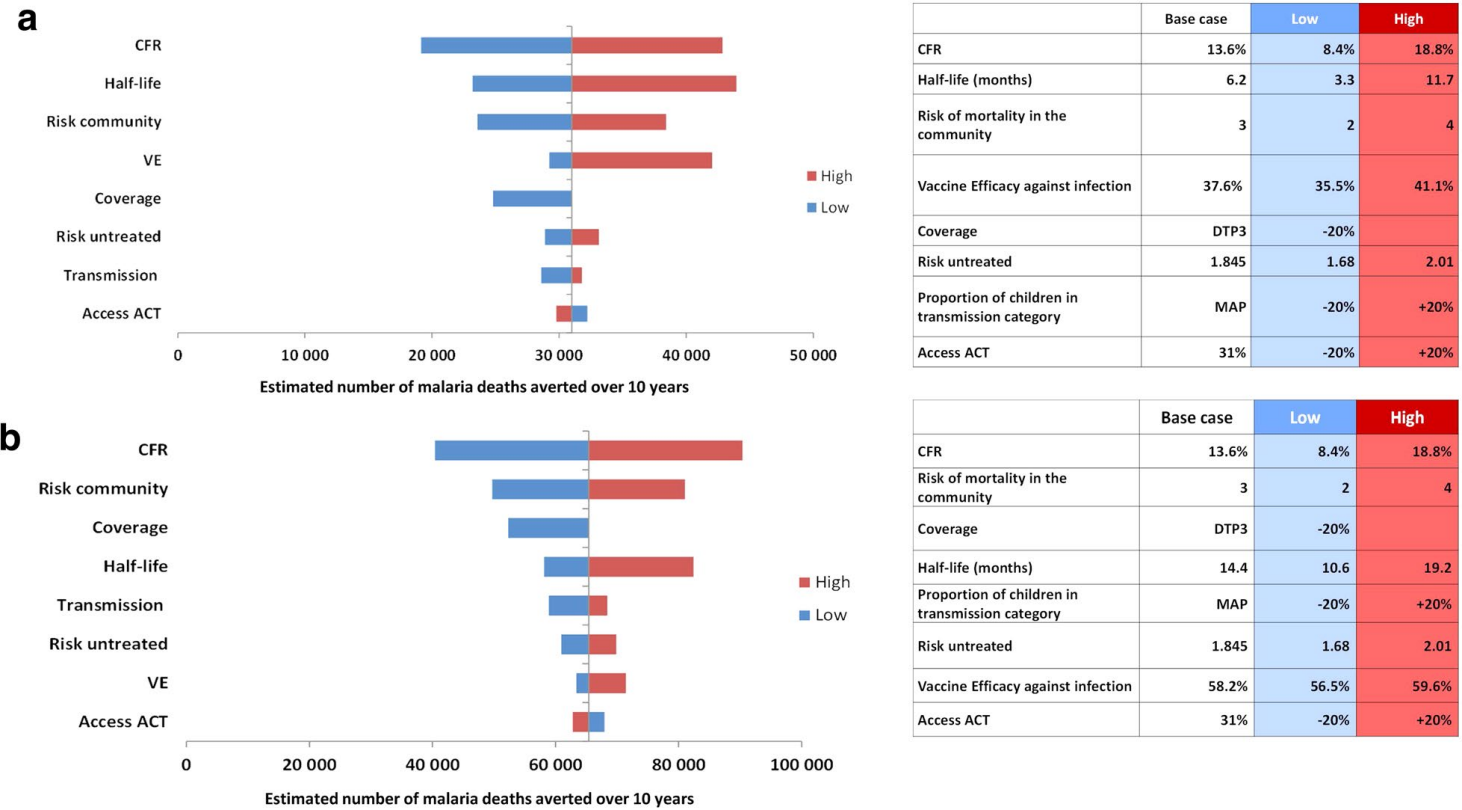
**a** Univariate sensitivity analysis on vaccination at 6, 10 and 14 weeks. Effect of varying each indicated input parameter to the upper and lower values shown on the total number of malaria deaths expected to be averted over 10 years by vaccination at age 6, 10 and 14 weeks CT, artemisinin-based combination therapy; *CFR* case-fatality rate, *DTP* diphtheria-tetanus-pertussis, *VE* vaccine efficacy, *MAP* Malaria Atlas Project. **b** Univariate sensitivity analysis on vaccination at 6, 7-and-a-half and 9 months. Effect of varying each indicated input parameter to the upper and lower values shown on the total number of malaria deaths expected to be averted over 10 years by vaccination at age 6, 7-and-a-half and 9 months. *ACT* artemisinin-based combination therapy, *CFR* case-fatality rate, *DTP* diphtheria-tetanus-pertussis, *VE* vaccine efficacy, *MAP* Malaria Atlas Project

Figure 5a, b present the results of the univariate sensitivity analysis for the total number of malaria deaths expected to be averted over 10 years by vaccination at age 6, 10 and 14 weeks or vaccination at age 6, 7-and-a-half and 9 months, respectively. The parameters with the largest impact on the results were waning of vaccine efficacy (decay) and the case-fatality rate.

## Discussion

This analysis applied 18-month follow-up data from a Phase III clinical trial to estimate the potential public health impact of the RTS,S candidate malaria vaccine in 42 malaria-endemic countries in sub-Saharan Africa using a cohort model. Two vaccination strategies were considered, with doses administered either at 6, 10 and 14 weeks (corresponding to adding the RTS,S vaccine to the EPI schedule) or at 6, 7-and-a-half and 9 months. Each strategy was compared with no vaccination to estimate the expected impact of RTS,S vaccination on averting clinical malaria cases, severe cases, malaria hospitalizations and malaria deaths in a birth cohort followed to the age of 10 years. Vaccination at age 6, 10 and 14 weeks would be expected to avert over five million cases of clinical malaria, 119,000 severe malaria cases, 98,600 malaria hospitalizations and 31,000 malaria deaths. Vaccination at age 6, 7-and-a-half and 9 months would be expected to avert almost 12.5 million cases of clinical malaria, 250,000 severe malaria cases, 208,000 malaria hospitalizations and 65,400 malaria deaths. The wide CIs reflect the combination of heterogeneity and uncertainty allowed for by the stochastic process in the model. The greatest uncertainty is around the impact on mortality.

After calibration, the model-predicted results closely matched the incidence of malaria observed in the control arm of the Phase III trial, and the published age distribution of malaria cases [14]. However, when extrapolating at the country level, the modelled estimates for the number of clinical malaria cases and malaria deaths were larger than the estimates for 2010 in the WHO World Malaria Report 2012, which estimated the number of clinical malaria cases in the WHO Africa region in all age groups at 174 million (upper bound 242 million), and the number of malaria deaths at 596,000 (upper bound 772,000) [26]. Possible reasons for the differences are

- The model is calibrated to the incidence of clinical malaria in the control arm of the Phase III trial based on the secondary case definition (parasite density of >0 and presence of fever). It may be possible that the secondary case definition would have captured cases of fever due to other illnesses and the malaria parasites observed were present as a co-morbidity rather than the primary cause. The primary case definition (parasite density of >5000 and presence of fever) would be more specific and lead to a reduction of estimates by 35 %.
- Estimated malaria episodes from WHO World Malaria report may not sufficiently adjust for under-reporting, although a correction factor is used;
- The model estimated malaria incidence based on reported proportion of children within categories of parasite prevalence from the MAP project. If this proportion varies over the year or the force of infection varies within a category, this is not captured by the model. Finally, the case-fatality rate for severe cases not treated in hospital is subject to a large uncertainty. An estimate from Thwing et al. [22] is applied in the model but this factor has a large impact on malaria mortality estimates (see Fig. 5a, b).

However it should be noted that clinical vaccine efficacy does not change with the primary or secondary case definition.

Another modelling estimate of 252 (95 % CI 171–353) million malaria episodes in sub-Saharan Africa for 2010 has been reported by Griffin et al. [27]. These estimates include the total population and suggest the proportion of cases that are in under-fives varies from above 60 % at high transmission to below 20 %. Hay et al. [28] have reported estimates of 271 million of malaria cases due *P. falciparum* in Africa in 2007.

Obtaining a realistic estimate of the number of severe malaria cases and malaria hospitalizations is important as these outcomes contribute to understanding the health burden of malaria. However, available data are limited and generally relate to populations with access to treatment. For example, the Phase III trial was conducted in settings where all patients had access to a high level of care. It was found that about half of the patients hospitalized with malaria met the case definition for severe malaria (parasite density <5000), which is similar to the value reported in a study of hospitalized patients in Tanzania, where 4261 patients of the 9337 with positive blood slides had severe disease (46 %) [20]. However, neither of these studies could provide data on the fraction of severe malaria cases that did not result in hospitalization, or on the risk of severe malaria developing in patients without access to treatment. The present analysis attempted to adjust for limited access to prompt and high-quality treatment for uncomplicated cases and limited access to hospitalization for severe cases.

Estimates of the potential impact of a pre-erythrocytic vaccine have been produced using other published malaria models developed by Imperial College London, the Swiss Tropical and Public Health Institute (Swiss TPH) and the Institute for Disease Modelling ([29–33]). These analyses have used a theoretical vaccine profile or calibrated the profile using results from a Phase II trial [29] and the 12-month follow-up data of the Phase III trial [31] of the RTS,S vaccine. Compared with the model presented in this study, the other models include the whole population (not just a birth cohort) and a dynamic malaria transmission process simulating vector population infectiousness and its interaction with the human host population. Therefore, the entomological inoculation rate is used as a measure of human host exposure, and can be affected by other interventions, such as insecticide-treated nets and indoor residual spraying. Those models include a mechanism of immunity acquisition acting on parasite density in individuals and reducing the probability of clinical disease, increasing tolerance to parasite and speeding-up parasite clearance. In the model described here, dynamic transmission is not included and no estimate for parasite density is made in individuals. Instead, parasite prevalence is used as a measure of exposure at the population level and a risk of infection is derived from trial incidence data. The immunity acquisition process against clinical disease depends on the number of previous infections in the individual. Treatment of malaria episodes is the only other intervention explicitly included in the model, because the effects of preventive vector-level interventions are assumed to be included in the estimates of parasite prevalence from MAP. Vaccine effect is introduced in a similar way to the other models, i.e., by reducing the initial risk of infection with no additional protection assumed. The definition of severe cases used in the present analysis is derived from the definition used in the Phase III trial, and differs from that used in an earlier analysis conducted with the Swiss TPH model [32]. However, the incidence of severe cases in the present analysis was similar to that reported by the Swiss TPH model [32].

One of the strengths of the present model is that it is simpler than the dynamic models and can be populated with readily available data, such as parasite prevalence and country-specific birth cohort data. The cohort approach used for the model helps to directly compare the results with the trial data. The model can thus be easily adapted and populated for specific countries. It is also relatively straightforward for decision-makers who are not experts in epidemiological modelling to understand and use. The present analysis has fitted the model to data from the most recent and large multicountry, multicentre trial on malaria vaccination, using the data available from the trial for the 18 months follow-up period, and may therefore be more representative of the current malaria epidemiological situation than models based on studies conducted during a period of very high transmission with no artemisinin-based combination therapy or long-lasting insecticide-treated nets.

The model has some limitations. First, as it is not a dynamic model it cannot take account of herd immunity. However, herd immunity is likely to be limited because of the small fraction of the population targeted by vaccination, the limited duration of protection and hence the small fraction of the parasite reservoir in human hosts which could be reached. Third, the model does not distinguish between different types of severe malaria, such as anaemia or cerebral malaria, which may have different implications for outcomes and resource use, although it does take account of the long-term neurological sequelae that can result from cerebral malaria. Fourth, it is assumed that the current levels of malaria transmission would remain stable over the period of analysis, although additional usage of long-lasting insecticide-treated nets and access to treatment are likely to further decrease malaria burden in the coming years. Conversely, the growing threat of resistance to insecticide and first-line treatment could lead to increasing malaria burden over that period.

A further area of work is to include seasonality in the model to improve the calibration using data from Nanoro trial site and impact estimate in seasonal settings, reflecting factors such as changes in temperature and/or rainfall.

The uncertainty around impact estimates is larger for severe malaria events and malaria deaths due to the trial design and stochastic variability. This leads to having lower bounds below zero on impact estimates for these outcomes, although it is a marginal effect in the 6, 7-and-a-half and 9 months schedule. It should be noted that the approach applied to characterize the uncertainty around the decay of vaccine-induced protection could potentially lead to overestimation of its variability. Structural uncertainty was assessed mainly around the natural immunity acquisition mechanism. The calibration process has been performed for model structures involving less immunity stages showing poorer fit on age-distribution data and severe malaria events. To test more immunity stages a functional form of immunity acquisition should be assumed in order to limit the number of parameters. Alternatively, transitions for the waning of acquired immunity were included but did not improve the fit. Vaccination has a different mechanism of action than existing malaria interventions. Rather than targeting the vector or directly acting on parasites in the blood, vaccination reduces the risk of infection by increasing immunity to the malaria circumsporozoite protein. The use of interventions with complementary effects may allow greater malaria control than a single intervention alone.

## Conclusions

Addition of RTS,S malaria vaccination to existing interventions with doses administered at either age 6, 10 and 14 weeks, or at age 6, 7-and-a-half and 9 months, would be expected to reduce substantially the incidence of clinical malaria, severe malaria, malaria hospitalizations and malaria deaths in children across 42 countries in sub-Saharan Africa, compared with no vaccination. There is a larger uncertainty for the estimates impact on of severe malaria cases and deaths.

## Additional files

10.1186/s12936-015-1046-z Probability density functions of the risk of infection parameter. Description: This figure shows the non-parametric probability density functions to represent the variability of the risk of infection parameter (q) in low, moderate and high transmission settings.
10.1186/s12936-015-1046-z Probability density functions of the probability of asymptomatic infection (a1–a6) and risk of severe malaria (f1–f6). Description: This figure shows the non-parametric probability density functions to represent the variability of the probability of asymptomatic infection (parameters a1–a6) and the variability of risk of developing severe malaria from a clinical malaria episode (parameters f1–f6).
10.1186/s12936-015-1046-z Estimated country-level impact of RTS,S vaccination. Estimated numbers of vaccinees and median and 95% confidence intervals for the number of clinical malaria cases, severe malaria cases, malaria hospitalizations and malaria deaths averted by RTS,S vaccination with doses administered either at 6, 10 and 14 weeks or at 6, 7.5 and 9 months. Outcomes are estimated for the annual birth cohort in each country, beginning in 2017 and following the cohort until the age of 10 years, assuming no change in malaria transmission.

## Abbreviations

DTP: Diphtheria-pertussis-tetanus
EPI: Expanded Programme on Immunization
MAP: Malaria Atlas Project
WHO: World Health Organization
DALY: Disability-adjusted life-year
CI: Confidence interval
PP: Parasite prevalence
Swiss TPH: Swiss Tropical and Public Health Institute
