# Supplementary figures and images for: Potential public health impact of RTS,S malaria candidate vaccine in sub-Saharan Africa: a modelling study
Source: Malar J. 2015 Dec 23;14:524. doi: 10.1186/s12936-015-1046-z (PMC4690265; doi:10.1186/s12936-015-1046-z)

Supplementary figure 1: Probability density functions of the risk of infection parameter (q)

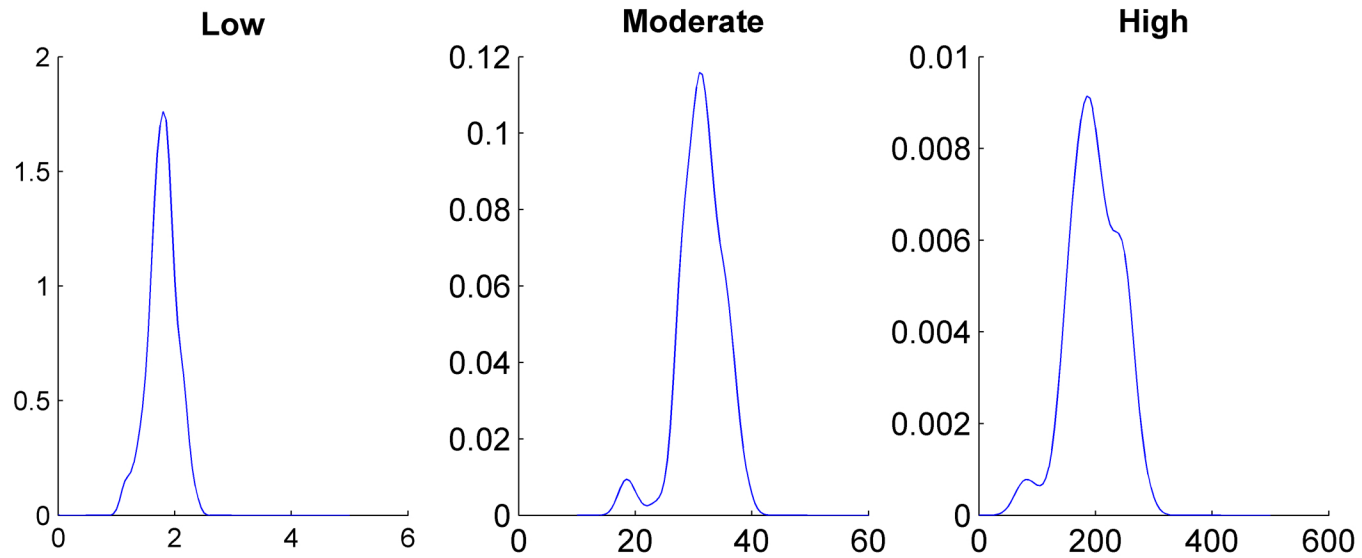

Supplement: Supplementary file 1 — 10.1186/s12936-015-1046-z Probability density functions of the risk of infection parameter. Description: This figure shows the non-parametric probability density functions to represent the variability of the risk of infection parameter (q) in low, moderate and high transmission settings. [file 12936_2015_1046_MOESM1_ESM.pdf]
