# Supplementary material for: Potential public health impact of RTS,S malaria candidate vaccine in sub-Saharan Africa: a modelling study
Source: Malar J. 2015 Dec 23;14:524. doi: 10.1186/s12936-015-1046-z (PMC4690265; doi:10.1186/s12936-015-1046-z)

Supplementary figure 2: Probability density functions of (A) the probability of asymptomatic infection (a1-a6) and (B) the risk of severe malaria (f1-f6)

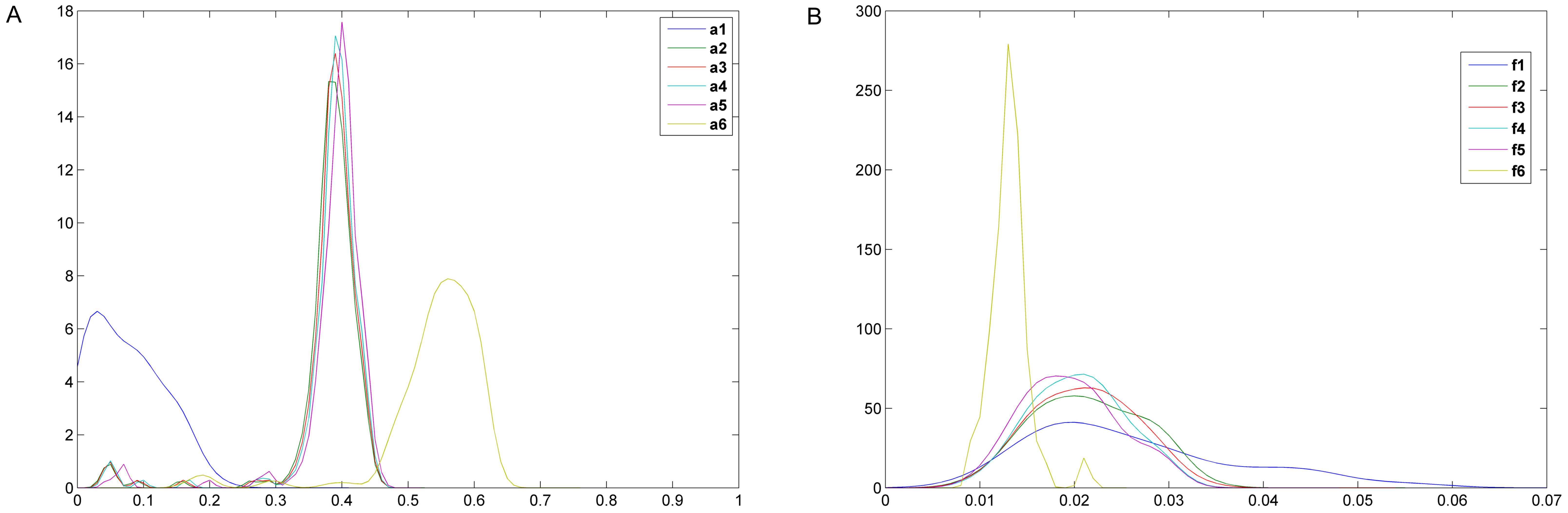

Supplement: Supplementary file 2 — 10.1186/s12936-015-1046-z Probability density functions of the probability of asymptomatic infection (a1–a6) and risk of severe malaria (f1–f6). Description: This figure shows the non-parametric probability density functions to represent the variability of the probability of asymptomatic infection (parameters a1–a6) and the variability of risk of developing severe malaria from a clinical malaria episode (parameters f1–f6). [file 12936_2015_1046_MOESM2_ESM.pdf]
