# Supplementary material for: Potential public health impact of RTS,S malaria candidate vaccine in sub-Saharan Africa: a modelling study
Source: Malar J. 2015 Dec 23;14:524. doi: 10.1186/s12936-015-1046-z (PMC4690265; doi:10.1186/s12936-015-1046-z)
Supplement: Supplementary file 3 — 10.1186/s12936-015-1046-z Estimated country-level impact of RTS,S vaccination. Estimated numbers of vaccinees and median and 95% confidence intervals for the number of clinical malaria cases, severe malaria cases, malaria hospitalizations and malaria deaths averted by RTS,S vaccination with doses administered either at 6, 10 and 14 weeks or at 6, 7.5 and 9 months. Outcomes are estimated for the annual birth cohort in each country, beginning in 2017 and following the cohort until the age of 10 years, assuming no change in malaria transmission. [file 12936_2015_1046_MOESM3_ESM.docx]

## Supplementary Table S1 - Estimated country-level impact of RTS,S vaccination

Estimated numbers of vaccinees and median and 95% confidence intervals for the number of clinical malaria cases, severe malaria cases, malaria hospitalisations and malaria deaths averted by RTS,S vaccination with doses administered either at 6, 10 and 14 weeks or at 6, 7.5 and 9 months. Outcomes are estimated for the annual birth cohort in each country, beginning in 2017 and following the cohort until the age of 10 years, assuming no change in malaria transmission.

|  |  | **Median (95% confidence interval)** | | | |
| --- | --- | --- | --- | --- | --- |
| **Country** | **Number of vaccinees** | **Clinical malaria cases averted** | **Severe malaria cases averted** | **Malaria hospitalisa-tions averted** | **Malaria deaths averted** |
| **Vaccination at 6, 10 and 14 weeks** | | | | | |
| Angola | 883 815 | 182 506 (31 134,338 052) | 4 715 (-7 805,13 807) | 3 917 (-6 484,11 471) | 1 231 (-2 038,3 605) |
| Benin | 339 942 | 105 695 (7 089,206 857) | 2 256 (-3 868,7 087) | 1 874 (-3 213,5 887) | 589 (-1 010,1 850) |
| Botswana | 45 326 | 718 (27,1 799) | 34 (-86,185) | 28 (-72,154) | 9 (-23,48) |
| Burkina Faso | 673 961 | 234 951 (2 090,477 517) | 4 182 (-6 846,14 515) | 3 474 (-5 688,12 059) | 1 092 (-1 788,3 790) |
| Burundi | 472 998 | 36 853 (9 245,65 635) | 1 318 (-2 750,3 855) | 1 095 (-2 284,3 202) | 344 (-718,1 007) |
| Cameroon | 578 287 | 177 765 (9 581,350 164) | 4 116 (-7 302,13 067) | 3 420 (-6 066,10 856) | 1 075 (-1 907,3 412) |
| Central African Republic | 88 435 | 30 995 (1 540,61 596) | 627 (-944,2 074) | 520 (-784,1 723) | 164 (-246,542) |
| Chad | 140 348 | 29 205 (6 803,51 275) | 967 (-1 726,2 484) | 803 (-1 434,2 064) | 252 (-451,649) |
| Comoros | 22 146 | 5 408 (471,10 380) | 138 (-274,410) | 115 (-227,340) | 36 (-71,107) |
| Congo | 157 239 | 39 495 (5 917,72 724) | 1 100 (-2 112,3 014) | 914 (-1 755,2 504) | 287 (-552,787) |
| Cote d'Ivoire | 486 629 | 170 207 (3 188,344 003) | 3 625 (-5 794,12 413) | 3 011 (-4 813,10 312) | 946 (-1 513,3 241) |
| Democratic Republic of Congo | 2 177 255 | 590 733 (77 960,1 114 625) | 13 943 (-22 087,41 080) | 11 583 (-18 349,34 128) | 3 641 (-5 767,10 727) |
| Djibouti | 20 719 | 13.1 (.6,32.6) | .6 (-1.5,3.3) | .5 (-1.3,2.8) | .2 (-.4,.9) |
| Equatorial Guinea | 9 332 | 3 116 (68,6 277) | 64 (-107,216) | 53 (-89,179) | 17 (-28,56) |
| Eritrea | 238 745 | 3 682 (141,9 216) | 173 (-442,948) | 144 (-367,787) | 45 (-115,248) |
| Ethiopia | 1 649 035 | 28 138 (1 354,69 943) | 1 317 (-3 290,7 167) | 1 094 (-2 734,5 954) | 344 (-859,1 872) |
| Gabon | 24 792 | 8 079 (-310,16 800) | 163 (-309,581) | 135 (-257,483) | 43 (-81,152) |
| Gambia | 83 914 | 8 242 (1 984,14 703) | 331 (-722,968) | 275 (-600,804) | 86 (-188,253) |
| Ghana | 739 857 | 180 785 (17 756,344 188) | 4 080 (-8 121,11 908) | 3 389 (-6 747,9 893) | 1 065 (-2 121,3 109) |
| Guinea | 270 523 | 66 938 (9 009,125 017) | 1 738 (-3 251,4 937) | 1 444 (-2 701,4 101) | 454 (-849,1 289) |
| Guinea-Bissau | 51 429 | 5 025 (1 360,8 860) | 176 (-357,509) | 146 (-297,423) | 46 (-93,133) |
| Kenya | 1 423 439 | 96 415 (12 032,189 548) | 2 805 (-6 042,9 841) | 2 330 (-5 020,8 176) | 732 (-1 578,2 570) |
| Liberia | 78 492 | 21 714 (1 254,42 426) | 455 (-887,1 388) | 378 (-737,1 153) | 119 (-232,363) |
| Madagascar | 776 464 | 157 953 (-530,323 372) | 3 963 (-7 939,13 791) | 3 292 (-6 596,11 457) | 1 035 (-2 073,3 601) |
| Malawi | 690 391 | 176 436 (16 338,336 861) | 4 239 (-8 418,12 405) | 3 521 (-6 993,10 306) | 1 107 (-2 198,3 239) |
| Mali | 580 147 | 177 889 (13 132,347 839) | 3 974 (-6 291,12 626) | 3 301 (-5 226,10 489) | 1 038 (-1 643,3 297) |
| Mauritania | 104 653 | 4 240 (736,8 197) | 150 (-330,521) | 124 (-274,433) | 39 (-86,136) |
| Mozambique | 811 535 | 212 858 (11 731,419 620) | 4 939 (-9 087,15 748) | 4 103 (-7 549,13 083) | 1 290 (-2 373,4 112) |
| Namibia | 50 668 | 2 505 (486,4 718) | 93 (-215,308) | 77 (-178,256) | 24 (-56,80) |
| Niger | 780 896 | 160 530 (35 975,280 977) | 4 836 (-9 296,12 200) | 4 018 (-7 723,10 136) | 1 263 (-2 427,3 186) |
| Nigeria | 3 641 124 | 1 137 914 (106 434,2 194 118) | 25 223 (-41 239,77 330) | 20 955 (-34 261,64 243) | 6 586 (-10 768,20 192) |
| Rwanda | 418 008 | 9 500 (1 128,20 944) | 442 (-1 071,2 024) | 367 (-889,1 681) | 115 (-280,528) |
| Sao Tome e Principe | 6 303 | 1 111 (279,1 883) | 38 (-82,92) | 31 (-68,77) | 10 (-21,24) |
| Senegal | 469 921 | 36 048 (7 275,67 343) | 1 477 (-3 337,4 803) | 1 227 (-2 772,3 990) | 386 (-871,1 254) |
| Sierra Leone | 190 148 | 57 886 (10 679,105 834) | 1 384 (-1 996,3 841) | 1 149 (-1 658,3 191) | 361 (-521,1 003) |
| Somalia | 206 530 | 4 481 (430,10 784) | 206 (-465,1 091) | 171 (-387,907) | 54 (-121,285) |
| Sudan: North | 1 241 322 | 26 440 (1 163,64 843) | 1 183 (-2 915,6 360) | 983 (-2 421,5 284) | 309 (-761,1 661) |
| Tanzania | 1 879 173 | 255 645 (37 085,478 220) | 6 484 (-13 984,19 374) | 5 387 (-11 618,16 096) | 1 693 (-3 652,5 059) |
| Togo | 208 817 | 59 822 (5 128,115 297) | 1 520 (-2 778,4 577) | 1 262 (-2 308,3 802) | 397 (-725,1 195) |
| Uganda | 1 462 572 | 411 047 (11 695,819 896) | 7 480 (-14 269,24 160) | 6 214 (-11 854,20 071) | 1 953 (-3 726,6 309) |
| Zambia | 565 745 | 72 297 (14 756,130 974) | 2 202 (-4 569,6 357) | 1 829 (-3 795,5 281) | 575 (-1 193,1 660) |
| Zimbabwe | 469 408 | 13 205 (1 251,30 802) | 583 (-1 389,2 930) | 484 (-1 154,2 434) | 152 (-363,765) |
| 42 countries in sub-Saharan Africa | 25 210 481 | 5 004 489 (474 867,9 684 160) | 118 736 (-214 795,372 995) | 98 642 (-178 445,309 873) | 31 004 (-56 087,97 396) |
| **Vaccination at 6, 7.5 and 9 months** | | | | | |
| Angola | 629 897 | 446 389 (306 564,520 855) | 9 491 (394,17 692) | 7 885 (327,14 698) | 2 478 (103,4 620) |
| Benin | 246 046 | 257 843 (162 426,304 414) | 4 934 (74,9 472) | 4 099 (61,7 869) | 1 288 (19,2 473) |
| Botswana | 33 302 | 2 157 (1 594,2 667) | 75 (-33,187) | 62 (-27,155) | 20 (-9,49) |
| Burkina Faso | 488 861 | 562 795 (327 910,669 793) | 9 985 (-54,19 791) | 8 295 (-45,16 441) | 2 607 (-14,5 168) |
| Burundi | 342 894 | 100 940 (80 707,117 033) | 2 387 (-64,4 484) | 1 983 (-53,3 725) | 623 (-17,1 171) |
| Cameroon | 420 491 | 439 000 (276 732,518 480) | 9 095 (97,17 504) | 7 556 (81,14 542) | 2 375 (25,4 571) |
| Central African Republic | 63 321 | 72 697 (42 945,86 187) | 1 439 (18,2 817) | 1 195 (15,2 340) | 376 (5,736) |
| Chad | 100 558 | 74 055 (55 270,85 547) | 1 777 (131,3 145) | 1 476 (109,2 613) | 464 (34,821) |
| Comoros | 16 130 | 13 920 (9 486,16 369) | 281 (,531) | 233 (,441) | 73 (,139) |
| Congo | 114 260 | 101 765 (72 561,118 630) | 2 133 (81,3 894) | 1 772 (67,3 235) | 557 (21,1 017) |
| Cote d'Ivoire | 351 106 | 405 684 (237 027,482 183) | 8 561 (-1,16 921) | 7 112 (-1,14 058) | 2 235 (0,4 418) |
| Democratic Republic of Congo | 1 551 474 | 1 414 342 (923 277,1 657 736) | 28 922 (1 270,54 286) | 24 028 (1 055,45 099) | 7 552 (332,14 175) |
| Djibouti | 15 030 | 39 (29,48) | 1 (-1,3) | 1.1 (-.5,2.8) | .4 (-.2,.9) |
| Equatorial Guinea | 6 747 | 7 524 (4 490,8 935) | 148 (-.3,292) | 123 (-.2,242) | 39 (-.1,76) |
| Eritrea | 175 723 | 11 063 (8 181,13 673) | 386 (-168,956) | 320 (-140,794) | 101 (-44,250) |
| Ethiopia | 1 208 046 | 84 176 (62 443,103 961) | 2 934 (-1 226,7 241) | 2 437 (-1 018,6 016) | 766 (-320,1 891) |
| Gabon | 18 200 | 20 143 (11 855,24 056) | 393 (-12,788) | 327 (-10,655) | 103 (-3,206) |
| Gambia | 61 390 | 23 015 (18 557,26 708) | 595 (-28,1 123) | 494 (-23,933) | 155 (-7,293) |
| Ghana | 541 400 | 468 774 (323 528,549 868) | 8 254 (82,15 470) | 6 857 (69,12 852) | 2 155 (22,4 040) |
| Guinea | 196 292 | 170 308 (118 446,199 012) | 3 465 (113,6 409) | 2 878 (94,5 324) | 905 (29,1 673) |
| Guinea-Bissau | 37 006 | 13 644 (10 983,15 793) | 315 (-5,589) | 262 (-4,490) | 82 (-1,154) |
| Kenya | 1 041 584 | 260 446 (189 489,307 495) | 5 641 (-636,11 453) | 4 686 (-528,9 515) | 1 473 (-166,2 991) |
| Liberia | 57 224 | 55 333 (36 451,65 286) | 956 (-2,1 827) | 795 (-2,1 518) | 250 (,477) |
| Madagascar | 570 468 | 403 044 (251 786,479 605) | 9 015 (-432,17 999) | 7 490 (-359,14 953) | 2 354 (-113,4 700) |
| Malawi | 505 894 | 456 733 (313 350,536 024) | 8 630 (94,16 181) | 7 169 (78,13 443) | 2 253 (25,4 225) |
| Mali | 417 802 | 423 870 (260 974,500 598) | 8 829 (195,17 001) | 7 335 (162,14 124) | 2 305 (51,4 439) |
| Mauritania | 75 943 | 11 715 (8 985,13 802) | 287 (-40,586) | 238 (-33,487) | 75 (-10,153) |
| Mozambique | 589 464 | 531 355 (340 053,627 790) | 10 741 (-30,20 772) | 8 923 (-25,17 257) | 2 805 (-8,5 424) |
| Namibia | 37 464 | 7 146 (5 684,8 376) | 173 (-23,346) | 143 (-19,287) | 45 (-6,90) |
| Niger | 567 258 | 420 117 (319 665,485 060) | 8 799 (583,15 475) | 7 310 (484,12 856) | 2 298 (152,4 041) |
| Nigeria | 2 624 558 | 2 749 346 (1 748 642,3 234 411) | 54 546 (1 758,103 606) | 45 315 (1 460,86 073) | 14 243 (459,27 054) |
| Rwanda | 307 435 | 27 768 (21 277,33 493) | 919 (-290,2 112) | 764 (-241,1 755) | 240 (-76,552) |
| Sao Tome e Principe | 4 637 | 3 098 (2 522,3 566) | 65 (2,113) | 54 (2,94) | 17 (1,30) |
| Senegal | 344 479 | 101 544 (80 459,118 862) | 2 741 (-315,5 441) | 2 277 (-262,4 520) | 716 (-82,1 421) |
| Sierra Leone | 134 258 | 135 615 (90 482,157 953) | 2 768 (212,5 071) | 2 299 (176,4 213) | 723 (55,1 324) |
| Somalia | 147 862 | 13 097 (9 839,16 115) | 453 (-157,1 103) | 376 (-130,916) | 118 (-41,288) |
| Sudan: North | 908 061 | 77 944 (57 049,96 064) | 2 642 (-1 059,6 476) | 2 195 (-879,5 380) | 690 (-276,1 691) |
| Tanzania | 1 382 820 | 693 506 (515 124,811 055) | 12 453 (-451,23 670) | 10 346 (-375,19 665) | 3 252 (-118,6 181) |
| Togo | 151 763 | 149 834 (98 654,176 177) | 3 204 (65,6 057) | 2 662 (54,5 032) | 837 (17,1 582) |
| Uganda | 1 072 051 | 1 038 791 (657 896,1 229 031) | 16 671 (-72,32 293) | 13 850 (-60,26 828) | 4 353 (-19,8 432) |
| Zambia | 412 334 | 195 061 (149 889,226 808) | 4 085 (-62,7 636) | 3 393 (-52,6 343) | 1 067 (-16,1 994) |
| Zimbabwe | 342 274 | 38 674 (29 132,47 207) | 1 260 (-442,3 005) | 1 047 (-368,2 497) | 329 (-116,785) |
| 42 countries in sub-Saharan Africa | 18 313 808 | 12 484 309 (8 242 412,14 696 728) | 250 448 (-433,481 819) | 208 064 (-359,400 280) | 65 397 (-113,125 813) |
